# Effects of nasal high flow on sympathovagal balance, sleep, and sleep-related breathing in patients with precapillary pulmonary hypertension

**DOI:** 10.1007/s11325-020-02159-1

**Published:** 2020-08-22

**Authors:** Jens Spiesshoefer, Britta Bannwitz, Michael Mohr, Simon Herkenrath, Winfried Randerath, Paolo Sciarrone, Christian Thiedemann, Hartmut Schneider, Andrew T. Braun, Michele Emdin, Claudio Passino, Michael Dreher, Matthias Boentert, Alberto Giannoni

**Affiliations:** 1 Department of Neurology with Institute for Translational Neurology, University of Muenster, Muenster, Germany; 2 Institute of Life Sciences, Scuola Superiore Sant’Anna, Piazza Martiri della Libertà, 33, 56127 Pisa, PI Italy; 3 Department of Medicine A, Hematology, Oncology and Pulmonary Medicine, University Hospital Muenster, Muenster, Germany; 4 Bethanien Hospital gGmbH Solingen, Solingen, Germany and Institute for Pneumology at the University of Cologne, Solingen, Germany; 5 Cardiology and Cardiovascular Medicine Division, Fondazione Toscana Gabriele Monasterio, National Research Council, CNR-Regione Toscana, Pisa, Italy; 6 Sleep Disorders Center, Bayview Hospital, School of Medicine, Johns Hopkins University, Baltimore, MD USA; 7 Division of Allergy, Pulmonary and Critical Care, Department of Medicine, University of Wisconsin, Madison, WI USA; 8 Department of Pneumology and Intensive Care Medicine, University Hospital RWTH, Aachen, Germany; 9 Department of Medicine, UKM Marienhospital, Steinfurt, Germany

**Keywords:** Pulmonary hypertension, Sympathovagal balance, Sleep, Sleep apnea, Nasal high flow

## Abstract

**Background:**

In precapillary pulmonary hypertension (PH), nasal high flow therapy (NHF) may favorably alter sympathovagal balance (SVB) and sleep-related breathing through washout of anatomical dead space and alleviation of obstructive sleep apnea (OSA) due to generation of positive airway pressure.

**Objectives:**

To investigate the effects of NHF on SVB, sleep, and OSA in patients with PH, and compare them with those of positive airway pressure therapy (PAP).

**Methods:**

Twelve patients with PH (Nice class I or IV) and confirmed OSA underwent full polysomnography, and noninvasive monitoring of SVB parameters (spectral analysis of heart rate, diastolic blood pressure variability). Study nights were randomly split into four 2-h segments with no treatment, PAP, NHF 20 L/min, or NHF 50 L/min. In-depth SVB analysis was conducted on 10-min epochs during daytime and stable N2 sleep at nighttime.

**Results:**

At daytime and compared with no treatment, NHF20 and NHF50 were associated with a flow-dependent increase in peripheral oxygen saturation but a shift in SVB towards increased sympathetic drive. At nighttime, NHF20 was associated with increased parasympathetic drive and improvements in sleep efficiency, but did not alter OSA severity. NHF50 was poorly tolerated. PAP therapy improved OSA but had heterogenous effects on SVB and neutral effects on sleep outcomes. Hemodynamic effects were neutral for all interventions.

**Conclusions:**

In sleeping PH patients with OSA NHF20 but not NHF50 leads to decreased sympathetic drive likely due to washout of anatomical dead space. NHF was not effective in lowering the apnea-hypopnoea index and NHF50 was poorly tolerated.

**Electronic supplementary material:**

The online version of this article (10.1007/s11325-020-02159-1) contains supplementary material, which is available to authorized users.

## Introduction

Alteration of autonomic nervous system function with increased sympathetic drive due to right ventricular dysfunction is frequently observed in patients with precapillary pulmonary hypertension (PH) and relates to both poor functional status and prognosis [1–5]. Furthermore, pharmacological treatment of PH may partially restore sympathovagal balance (SVB), as documented by a 20% decrease in heart rate variability (HRV; a surrogate marker of sympathetic drive) after sildenafil administration [6]. Electrical stimulation of the vagal nerve in a rat model of PH was shown to decrease sympathetic drive (again by ~ 20%), translating into a reduction in mean pulmonary arterial pressure and amelioration of pulmonary vascular remodeling, right ventricular function, and survival [7].

Sleep-disordered breathing (SDB), especially obstructive sleep apnea (OSA), is highly prevalent in patients with PH, affecting as many as one in four patients and may lead to an undesirable increase in sympathetic drive [8–10]. The therapeutic gold standard for OSA is the application of positive airway pressure (PAP) [11]. In patients with normal cardiac function without PH, PAP therapy has been shown to improve sleep and decrease sympathetic drive through reversal of obstructions that are otherwise associated with sympathetic surges, negative intrathoracic pressure, and arousals [12–14]. However, in patients with heart failure and reduced ejection fraction (HFrEF), PAP treatment of SDB has been shown to have hypotensive effects [15]. These might be deleterious in PH because right ventricular systolic dysfunction makes patients dependent on sufficient venous return and right ventricular preload, both of which are reduced by the increase in intrathoracic pressure associated with PAP [15]. This could outweigh the positive effects derived from reversal of upper airway obstructions, with overall neutral effects or undesired increases in sympathetic drive [15, 16]. Nasal high flow therapy (NHF) has been shown to build up flow-dependent positive airway pressure leading to a reduction of OSA, at least in children [17–19], but it remains to be determined whether the PAP generated by NHF is sufficient to impact on OSA severity and whether this would be accompanied by a beneficial decrease in sympathetic drive in patients with PH [17–19].

NHF may have favorable effects on nocturnal ventilation and sympathetic drive in patients with PH even in the absence of effective sleep apnea reduction. Increased dead space ventilation is known to play an important pathophysiological role in PH [20], leading to overcompensatory chronic hyperventilation. It has been previously shown that such “wasted ventilation” contributes to continued distress in PH patients and correlates to worse prognosis [21]. By using scintigraphy with ^81m^Krypton (^81m^Kr) gas in healthy volunteers and tracheotomized patients, Möller and coworkers showed that NHF (15, 30, and 45 L/min) decreased the ^81m^Kr gas clearance half-time in the upper airway (volumes calculated by magnetic resonance imaging [MRI]), including nasal cavities, the pharynx, and the trachea, resulting in a flow- and time-dependent decrease in dead space and rebreathing, thereby improving ventilatory efficiency [22]. Recently, NHF was shown to reduce peripheral vascular sympathetic activity during sleep in patients with chronic obstructive pulmonary disease, which might also be attributed to favorable effects on SVB by reduction of dead space ventilation [23].

Given that sympathetic drive is markedly increased in PH, we hypothesized that nocturnal administration of NHF in patients with PH and SDB would improve SVB during sleep, alleviate OSA, and decrease daytime sympathetic drive.

## Materials and methods

### Study design

Patients with Nice class I pulmonary arterial hypertension or class IV chronic thromboembolic PH (collectively referred to as precapillary PH) were consecutively recruited from May 2018 to April 2019. The study protocol conforms to the 1975 Declaration of Helsinki and was approved by the institution’s human research committee (Ethikkommission der Ärztekammer Westfalen Lippe und der Medizinischen Fakultät der Westfälischen Wilhelms-Universität Münster). All participants gave written informed consent to participate in the study. The project has been registered and updated prospectively under the German Clinical Trials Registry (drks.de Identifier: DRKS00013907 and DRKS00013907).

### Study participants

Both males and females participated in the study. Participants had to be at least 18 years of age and able to consent. Diagnosis of precapillary PH was established according to the most recent Guidelines of the European Society of Cardiology (ESC) [24]. Inclusion criteria comprised diagnosis of precapillary PH at least 12 weeks before recruitment and no hospitalization for heart failure (HF) within 4 weeks prior to enrolment. Patients had to have had optimal medical therapy in accordance with the most recent ESC guidelines [24] with combination therapy and no change in medication in the last 4 weeks. If patients were not treated with combination therapy, the reason was documented. PH patients had to be diagnosed with sleep-disordered breathing (SDB) with predominant OSA (apnea-hypopnea index ≥ 10/h; ≥ 50% of apneic events being obstructive).

Exclusion criteria were as follows: secondary (postcapillary) pulmonary hypertension (mean pulmonary arterial hypertension > 25 mm Hg; PCWP > 15 mm Hg); chronic obstructive pulmonary disease; insulin-dependent diabetes; severe renal impairment; severe neurological preexisting conditions including prior stroke; intake of opioids; severe mental disease. Detailed inclusion and exclusion patient criteria are available in the German Clinical Trials Registry ID DRKS00013906 and DRKS00013907.

### Baseline assessments

**Fig. 1:**
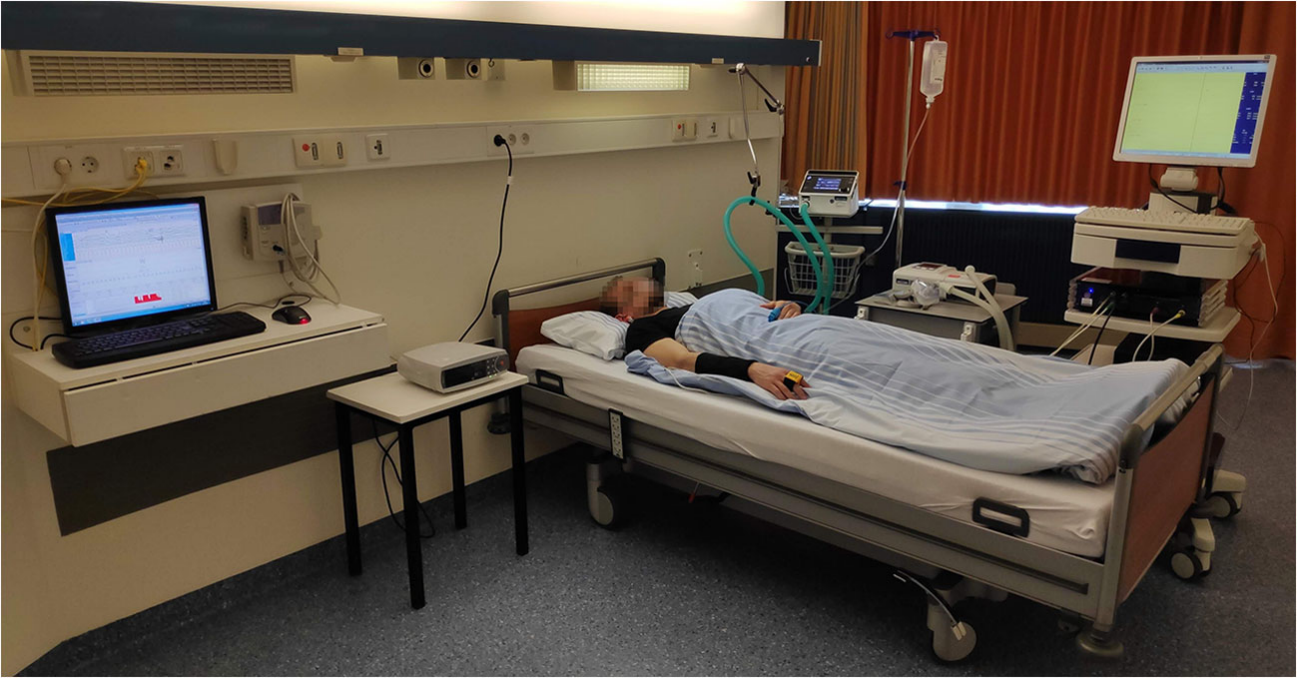
Experimental setup showing a patient connected to the respiratory, polysomnography, and noninvasive autonomic nervous system monitoring systems

All study participants underwent standard 2-dimensional Doppler echocardiography (LOGIQ S8-XD clear^TM^, GE Healthcare, London, United Kingdom) [25, 26]. Patients with PH also underwent a 1.5 Tesla MRI scan (Ingenia^TM^, Philips Healthcare, Best, The Netherlands) to quantify right ventricular systolic function [27], which was classified as normal or impaired based on age- and gender-specific reference values [27]. Patients also underwent noninvasive hemodynamic and respiratory monitoring in the afternoon in the sleep laboratory, and then spent a diagnostic night (night 1) and a study night (night 2) during attended polysomnography (PSG), as explained below (Fig. 1).

### Study protocol: effects of NHF at nighttime

To ascertain SDB with predominant OSA, patients underwent full diagnostic PSG (Somno HD^TM^, Somnomedics, Randersacker, Germany) in the academic sleep laboratory at Münster University Hospital. Respiratory recordings included nasal airflow (Ternimed^TM^, Bielefeld, Germany), thoracic and abdominal effort, and peripheral oxygen saturation (SpO_2_). Respiratory events were scored according to the guidelines of the American Academy of Sleep Medicine (AASM) [28]. Apnea was defined as a reduction in nasal airflow by > 90% of baseline for > 90% of the event’s duration and > 10 s. Apneas were classified as obstructive, if there was a continued or increased inspiratory effort throughout the entire period of absent airflow. If this was not the case, apneas were classified as central. Hypopnea was defined as *a* > 30% fall from baseline in airflow signal for 90% of the event’s duration of at least 10 s and 3% desaturation from the preevent baseline. The oxygen desaturation index (ODI) was based on decreases in peripheral oxygen saturation of 3% at least.

Sleep stages were recorded and analyzed using a twelve-lead electroencephalography (EEG) in accordance with the most recent AASM guidelines [28–35].

During the next night, full PSG was combined with transcutaneous carbon dioxide (CO_2_) monitoring (SenTec, Therwil, Switzerland). The night was split into four parts of 2-h duration each. The first part was recorded without treatment of SDB (with some patients requiring oxygen), then PAP, NHF at 20 L/min (NHF20), and NHF 50 L/min (NHF50) were given for 2 h each in a randomized order. NHF (PrismaVent 50^TM^, Löwenstein Medical, Bad Ems, Germany) was administered using a nasal cannula (Optiflow^TM^, Fisher & Paykel, Schorndorf, Germany), and flow rates were chosen based on previous studies showing differential effects on ventilation and oxygenation [17, 20, 21]. Automatic PAP (5–12 cm H_2_O) was delivered through a nasal mask. Noninvasive hemodynamic monitoring (detailed below) was performed throughout the second night. All measurements were fully attended (B. B. and J. S.), and sleep stages, respiration, and SVB were evaluated online and on a “beat-to-beat” basis. Four 10-min sleep segments for each patient (one each from the NT, PAP, NHF20, and NHF50 treatment periods) were chosen by visual identification. Each segment had to be characterized by stable N2 (nonrapid eye movement stage 2) sleep and sinus rhythm with fewer than 5% ectopic beats.

### Study protocol: effects of NHF during daytime

The acute daytime effects of NHF therapy on SVB and hemodynamics were assessed in the late afternoon with the patient awake. Initially, subjects were asked to breathe spontaneously for 10 min to obtain baseline parameters. Thereafter, patients were exposed to 10 min each of NHF20 and NHF50 in a random order, separated by periods of spontaneous breathing until heart rate, blood pressure, transcutaneous carbon dioxide, and oxygen saturation had returned to baseline. Patients were asked to rate their subjective well-being at baseline and after each NHF intervention using a visual analogue scale ranging from 0 to 10 (0 = worst overall wellbeing imaginable; 10 = best wellbeing imaginable).

### Noninvasive hemodynamic monitoring

SVB and hemodynamics were measured using a noninvasive monitor device (Task Force Monitor^TM^, CNSystems, Graz, Austria), as previously validated [29–31]. For assessment of SVB, diastolic blood pressure variability (BPV) and heart rate variability (HRV) were analyzed using the continuous noninvasive arterial blood pressure signal (CNAP^TM^ technology, sampling rate 100 Hz) and a 3-lead electrocardiogram (sampling rate 1000 Hz), both implemented in the Task Force Monitor^TM^. Diastolic BPV (measured in mm Hg^2^) and HRV (i.e., variability in RR intervals measured in ms^2^) were both continuously recorded and then normalized for total power spectra with the resulting unit being %. Data were computed by frequency domain analysis (adaptive autoregressive parameter model) and presented as the high frequency component (HF: 0.15–0.40 Hz), low frequency component (LF: 0.04–0.15 Hz), and relative ratio (LF/HF) for both HRV and diastolic BPV [29–32]. For both measurements, a higher ratio indicates increased sympathetic drive because the LF component is believed to mainly reflect sympathetic drive, and the HF component is believed to exclusively reflect parasympathetic drive [29–31, 33].

Baroreceptor reflex sensitivity (BRS) was measured using the sequence method [29–32, 34]. The time-constant of this stimulus-response relationship primarily reflects, particularly with respect to the upsequences, vagal but not sympathetic responsiveness [29–32, 34].

Hemodynamic measurements included beat-to-beat systolic (sBP) and diastolic (dBP) blood pressure; these were recorded and validated against periodic measurements obtained every 15 min by oscillometric recording from the upper contralateral arm. Transthoracic impedance measurements were used to estimate cardiac stroke volume index (SVI), cardiac index (CI), and systemic vascular resistance (SVR), from which the total peripheral resistance index (TPRI) was calculated (mean blood pressure divided by CI). Bioimpedance-based measurements have been previously validated against invasive hemodynamic monitoring in the catheter laboratory [29–31].

All signals were simultaneously acquired and displayed in real time using personal computer running DOMINO 2.9.0 software.

### Statistical analysis

All analyses were performed using Sigma Plot^TM^ software (Version 13.0, Systat Software GmbH, Erkrath, Germany). Assuming a two-sided significance level of 0.05 (alpha) and 80% power (beta), a sample size of twelve patients per group was calculated to allow detection of a 20% change in the LF/HF ratio of HRV [6, 7]. Values for the mean and standard deviation of the HRV LF/HF ratio during 10 min of N2 sleep were obtained from previous preliminary data. Furthermore, it was also known from our previous measurements that intraindividual variation in the LF/HF ratio component of HRV during N2 sleep is ~ 5% at most.

Results were expressed as mean and standard deviation for continuous variables with a normal distribution, and median and interquartile range for continuous variables with a skewed distribution. Categorical variables were expressed as percentages unless otherwise specified. Respiratory parameters, SVB, and hemodynamic measures recorded during the different interventions were compared with baseline values using a paired *t* test or Wilcoxon rank sum test, as appropriate. For all tests a *p* value ≤ 0.05 was considered statistically significant.

## Results

A total of twelve patients with PH were enrolled in the study (Table 1). Two out of 12 patients had been receiving nocturnal PAP therapy prior to enrolment. We did not exclude these patients since long-term adaptation to mask-based treatment could be assumed in these individuals. PH etiology was connective tissue disease (*n* = 8), idiopathic pulmonary arterial hypertension (*n* = 1), drug-induced PH (*n* = 1), and chronic thromboembolic pulmonary hypertension (*n* = 2). Most patients were mildly symptomatic, although 33% were in New York Heart Association class III or IV. All patients had preserved left ventricular ejection fraction on presentation, but four showed right ventricular dysfunction on cardiac MRI scans. SDB was moderate to severe, with frequent oxygen desaturations (as shown by the oxygen desaturation index [ODI]) on the background of chronic hypoxemia (reflected by the time spent with oxygen saturation below 90% [T90]). The majority of patients (10/12) were receiving targeted therapy, as recommended for first-line treatment [24].

**Table 1:** Demographic and clinical characteristics of the study population at baseline

	Patients (*n* = 12)
Male, *n* (%)	5 (41.7)
Age, years	68.4 ± 11.1
BMI, kg/m²	30.5 ± 3.5
BSA, m^2^	2.0 ± 0.2
NYHA class I, *n* (%)	1 (8.3)
NYHA class II, *n* (%)	7 (58.3)
NYHA class III, *n* (%)	3 (25)
NYHA class IV, *n* (%)	1 (8.3)
LVEF, %	60.9 ± 7.0
Impaired LVEF, *n* (%)	0 (0)
RVEF, %	53.5 ± 12.0
Impaired RVEF, *n* (%)	3 (30)
AHI, /h	21.5 (12.2–46.2)
AHI > 10/h, *n* (%)	12 (100)
AI, /h	8.2 (1.5–19.7)
cAI, /h	0.6 (0.0–6.8)
oAI, /h	4.5 (1.4–12.3)
HI, /h	11.5 (4.2–26.4)
T < 90%, min	166.2 ± 102.1
Minimum oxygen saturation, %	72.4 ± 10.4
Mean oxygen saturation, %	89.6 ± 2.7
tcCO_2_, mmHg	39.2 ± 5.8
ODI, /h	14.4 (10.8-32.2)
Medication, *n* (%)	10 (83.3)
Phosphodiesterase inhibitor	5 (41.7)
Direct cyclic guanylate cyclase stimulator	3 (25)
Endothelin receptor antagonist	5 (41.7)
Prostacyclin analog	0 (0)

Values are mean ± standard deviation, median (interquartile range), or number of patients (%)

*BMI*, Body mass index; *BSA*, body surface area; *NYHA*, New York Heart Association; *LVEF*, left ventricular ejection fraction; *RVEF*, right ventricular ejection fraction; *AHI*, apnea-hypopnea index; *AI*, apnea index; *cAI*, central apnea index; *oAI*, obstructive apnea index; *HI*, hypopnea index; *tcCO*_*2*_; transcutaneous carbon dioxide pressure; *ODI*, oxygen desaturation index; Cardiac MRI data were obtained in 10/12 patients (2 patients did not consent to the procedure)

### Impact of NHF20 on sympathovagal balance, sleep, and sleep apnea

**Fig. 2:**
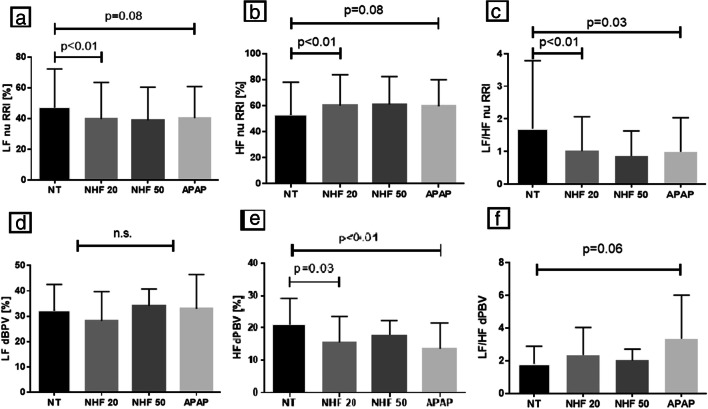
Impact of nasal high flow therapy at 20 L/min (NHF 20), nasal high flow therapy at 50 L/min (NHF50), and automatically titrating positive airway pressure (APAP) compared with no treatment (NT) on sympathovagal balance. dBP, diastolic blood pressure; HF, high frequency component; LF, low frequency component; LF/HF, low frequency/high frequency component ratio; nu RRI, heart rate variability normalized for total power spectra (normalized units). Bars show mean values with standard deviation (lines) derived from segments of 10 min taken from stable N2 sleep with sinus rhythm

**Fig. 3:**
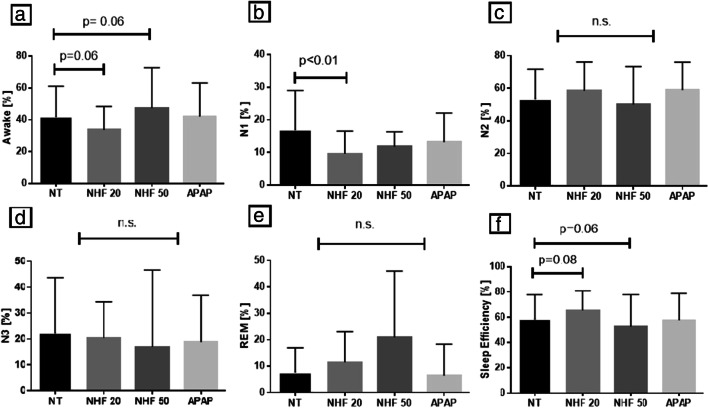
Impact of nasal high flow therapy at 20 L/min (NHF 20), nasal high flow therapy at 50 L/min (NHF50) and automatically titrating positive airway pressure (APAP) compared with no treatment (NT) during periods of awake (**a**), N1 sleep (**b**), N2 sleep (**c**), N3 sleep (**d**), rapid eye movement (REM) sleep (**e**), and overall sleep efficiency (**f**). Bars show mean values with standard deviation (lines)

**Fig. 4:**
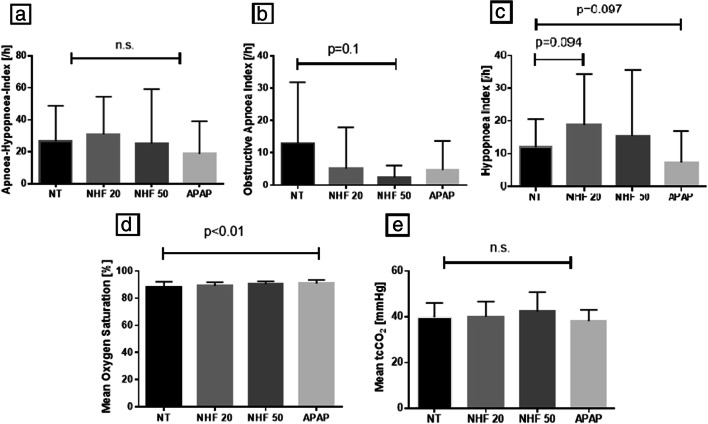
Impact of nasal high flow therapy at 20 L/min (NHF 20), nasal high flow therapy at 50 L/min (NHF50) and automatically titrating positive airway pressure (APAP) compared with no treatment (NT) on sleep-related breathing parameters: apnea-hypopnea index (**a**), obstructive apnea index (**b**), hypopnea index (**c**), mean oxygen saturation (**d**), mean trans cutaneous carbon dioxide (tcCO_2_) (**e**). Bars show mean values with standard deviation (lines)

Compared with no treatment, hemodynamic parameters remained unchanged during NHF20. There was a shift towards predominance of parasympathetic drive over sympathetic drive during N2 sleep, shown by an increase (~ 15%) in the HRV HF component and a decrease (by ~ 25%) in the HRV LF component, and a decrease (by ~ 40%) in the HRV LF/HF ratio with delivery of NHF20 during N2 sleep (Table 2 and Fig. 2). There was also a trend towards better sleep efficiency during use of NHF20 (from ~ 58 to ~ 65% (*p* = 0.076) (Table 3 and Fig. 3). NHF20 had no significant effects on the severity of SDB (Table 3 and Fig. 4).

**Table 2:** Effect of positive airway pressure and nasal high flow therapy on sympathovagal balance and hemodynamics during N2 sleep

	No treatment (*n* = 12)	NHF20 (*n* = 12)	*P* value^+^	NHF50 (*n* = 6)	*P* value^+^	APAP (*n* = 12)	*P* value^+^
Sympathovagal balance parameters							
HFnuRRI, %	52.8 ± 25.2	60.1 ± 23.7	*0.005*	61.0 ± 21.4	0.362	59.6 ± 20.4	*0.079*
LFnuRRI, %	47.2 ± 25.2	39.9 ± 23.7	*0.005*	39.1 ± 21.4	0.362	40.5 ± 20.4	*0.079*
LF/HF nu RRI	1.7 ± 2.1	1.0 ± 1.0	*0.009*	0.9 ± 0.8	0.193	1.0 ± 1.0	*0.034*
HFnudBPV, %	21.0 ± 8.1	15.4 ± 8.1	*0.028*	17.9 ± 4.5	0.094	13.6 ± 8.0	*0.004*
LFnudBPV, %	32.2 ± 10.4	28.2 ± 11.6	0.247	34.1 ± 6.6	0.290	33.0 ± 13.5	0.846
LF/HF nu dBPV	1.8 ± 1.1	2.3 ± 1.7	0.129	2.0 ± 0.7	0.355	3.3 ± 2.7	*0.060*
BRS slope*							
Up event counts	19.0 (17.0-26.0)	21.0 (17.0-39.0)	0.637	11.0 (9.3-12.8)	*0.037*	16.0 (8.0-24.0)	0.389
Up events, ms/mmHg	6.0 (5.6-14.4)	8.2 (5.9-14.5)	0.570	14.5 (9.3-27.6)	0.923	13.4 (4.6-17.7)	0.652
Down event count	17.5 (16.3-30.3)	25.0 (11.3-28.0)	0.978	7.0 (6.0-13.0)	0.248	18.0 (7.8-24.0)	0.622
Down events, ms/mmHg	7.6 (5.9-13.4)	8.7 (7.5-12.1)	0.557	11.0 (4.2-12.4)	0.862	10.7 (5.7-17.1)	0.084
Hemodynamic parameters							
Heart rate, min^−1^	69.7 ± 9.7	65.9 ± 9.4	*0.021*	64.3 ± 12.2	0.155	66.5 ± 9.0	*0.001*
Systolic BP, mmHg	105.9 ± 13.3	99.8 ± 8.7	0.242	104.9 ± 14.9	0.687	106.8 ± 10.4	0.865
Diastolic BP, mmHg	61.5 ± 12.1	59.7 ± 7.0	0.655	62.6 ± 6.0	0.730	62.9 ± 9.8	0.746
Stroke volume index, mL/m²	25.7 ± 5.0	24.9 ± 4.5	0.164	23.3 ± 3.5	0.391	26.5 ± 5.3	0.910
Cardiac index, L/min/ m²	1.8 ± 0.4	1.6 ± 0.4	0.165	1.5 ± 0.4	0.191	1.8 ± 0.4	0.193
TPRI, dyne·s m² cm^−5^	3680.9 ± 1198.6	3808.8 ± 1133.4	0.412	4233.5 ± 660.0	0.674	3794.3 ± 1106.1	0.204
Respiratory parameters							
Mean SpO_2_, %	90.5 ± 3.3	91.0 ± 2.3	0.324	92.0 ± 1.9	0.586	91.5 ± 2.7	0.349
tcCO_2_, mmHg**	38.5 ± 3.7^°^	39.0 ± 4.6^°^	0.384	39.6 ± 5.8	0.650	38.1 ± 5.7^°^	0.641

Values are mean ± standard deviation or median (interquartile range) for segment of 10 min in duration taken from stable N2 sleep with sinus rhythm. Italicizised data indicates that the *p* value is below 0.10

*APAP*, Automatic positive airway pressure; *NHF20*, nasal high flow therapy at 20 L/min; *NHF50*, nasal high flow therapy at 50 L/min; *PH*, precapillary pulmonary hypertension; *BRS Slope*, slope of baroreceptor reflex sensitivity (up events and down events); *HFnudBPV*, high frequency component of diastolic blood pressure variability; *HFnuRRI*, high frequency component of heart rate variability; *LFnudBPV*, low frequency component of diastolic blood pressure variability; *LF/HF dBPV*, relative ratio of low frequency and high frequency component of diastolic blood pressure variability; *LFnuRRI*, low frequency component of heart rate variability; *LF/HF RRI*, relative ratio of low frequency and high frequency component of heart rate variability; *nu*, normalized units (normalized for total power spectra); for both measures a higher ratio reflects increased sympathetic drive as LF component reflects sympathetic drive and HF component (of both HRV and dBPV) reflects parasympathetic drive [24–26, 29]; TPRI, total peripheral resistance index

^**+**^For comparison versus baseline. *Due to the low number of events in a subset of patients analysis of BRS was performed in 9/12 patients with no treatment and NHF20, and in 4/6 with NHF50, for up events; and in 10/12 patients with no treatment and NHF20, and in 5/6 with NHF50 for down-events. **Due to artifacts in capnometry recordings, analysis was performed in 9/12 patients with no treatment, NHF20 and APAP, and in 3/6 patients with NHF50

**Table 3:** Acute effects of positive airway pressure and nasal high flow therapy on obstructive sleep apnea and sleep

	No treatment (*n* = 12)	NHF20 (*n* = 12)	*P* value^+^	NHF50 (*n* = 6)	*P* value^+^	APAP (*n* = 12)	*P* value^+^
Duration of intervention, min (%TIB of the entire night)	136.8 ± 35.1 (30.3 ± 7.8)	130.2 ± 36.8 (28.7 ± 7.5)	0.569 (0.569)	87.2 ± 30.2 (19.5 ± 6.2)	0.142 **(**0.135)	133.2 ± 47.2 (29.4 ± 10.0)	0.856 (0.837)
Duration asleep (TST), min (% TIB of the intervention period)	69.5 (54.9–96.6) (58.4 ± 19.4)	83.0 (71.3–92.6) (65.4 ± 15.5)	0.266 (*0.075*)	45.5 (27.8–62.9) (52.6 ± 25.3)	*0.069 (0.060)*	71.0 (56.3–93.5) (58.0 ± 21.1)	0.770 (0.953)
Supine (% TST)	76.8 (31.9–100.0)	97.6 (49.0–100.0)	0.313	56.7 (12.7–91.2)	0.500	85.0 (50.9–100.0)	0.520
Sleep							
Awake, min (% of the intervention period)	51.5 (39.1–81.8) (48.4 (29.8–56.8))	41.8 (30.5–65.9) (32.2 (23.7–45.8))	*0.077 (0.061)*	37.8 (26.6–56.4) (47.3 (37.5–65.1)))	0.985 (*0.060*)	60.0 (33.1–79.8) (40.2 (29.3–53.5))	0.869 (0.953)
TST, min (% of the intervention period)	69.5 (54.9–96.6) (58.4 ± 19.4)	83.0 (71.3–92.6) (65.4 ± 15.5)	0.266 (*0.075*)	45.5 (27.8–62.9) (52.6 ± 25.3)	*0.069 (0.060)*	71.0 (56.3–93.5) (58.0 ± 21.1)	0.770 (0.953)
N1, min (% of the intervention period)	8.8 (7.3–14.1) (13.6 (7.7–23.0))	6.3 (4.5–10.8) (7.7 (5.8–11.1))	*0.004 (0.005)*	5.0 (3.1–6.1) (11.9 (8.4–14.2))	0.127 (0.902)	8.5 (4.8–14.4) (9.4 (6.7–19.8))	0.466 (0.296)
N2, min (% of the intervention period)	36.0 (25.0–53.4) (53.1 (37.4–60.9))	46.5 (34.0–59.4) (60.5 (48.7–69.0))	0.233 (0.487)	20.3 (14.0–31.0) (55.9 (33.2–60.3))	*0.057* (0.837)	40.5 (29.8–52.1) (55.1 (43.1–75.4))	0.813 (0.469)
N3, min (% of the intervention period)	11.3 (0.8–29.5) (18.4 (1.6–42.5))	14.3 (8.5–24.0) (22.9 (11.3–28.8))	0.610 (0.809)	0.0 (0.0–13.9) (0.0 (0.0–22.7))	0.313 (0.540)	14.0 (0.8–24.1) (15.4 (3.5–28.4))	0.610 (0.673)
REM, min (% of the intervention period)	0.5 (0.0–15.3) (0.9 (0.0–15.8))	10.3 (0.0–17.8) (10.7 (0.0–20.1))	0.668 (0.436)	4.8 (0.0–10.3) (15.9 (0.0–33.5))	0.445 (0.295)	0.0 (0.0–10.0) (0.0 (0.0–8.9))	0.859 (0.806)
Sleep efficiency, %	58.3 ± 19.6	65.4 ± 15.5	*0.076*	52.6 ± 25.3	*0.059*	57.8 ± 21.0	0.941
Arousal index, /h	19.0 (12.3–30.0)	17.4 (14.8–25.2)	0.981	10.3 (6.5–14.6)	0.438	17.5 (10.1–22.8)	0.453
Respiratory-related arousals, /h	5.7 (2.7–14.3)	11.1 (7.8–13.9)	0.580	2.8 (0.5–23.1)	0.604	3.9 (2.0–13.0)	0.325
PLM-related arousals, /h	5.4 (0.0–16.1)	0.7 (0.0–6.2)	0.098	3.5 (0.0–8.4)	0.810	0.6 (0.0–5.2)	0.149
Sleep-related breathing							
AHI, /h	20.8 (9.6–38.4)	28.5 (17.0–46.8)	0.465	6.5 (2.4–49.0)	0.741	6.3 (4.9–32.4)	0.254
AI, /h	6.9 (0.9–19.4)	1.1 (0.5–7.1)	0.667	2.4 (0.2–12.7)	0.687	0.5 (0.0–22.5)	0.557
cAI, /h	0.0 (0.0–0.3)	0.0 (0.0–0.1)	0.813	1.9 (0.0–7.6)	0.138	0.4 (0.0–9.0)	0.156
oAI, /h	6.9 (0.5–14.0)	0.6 (0.0–2.4)	0.266	0.0 (0.0–5.0)	0.125	0.0 (0.0–3.1)	0.322
HI, /h	9.2 (6.6–17.7)	19.7 (3.4–30.9)	*0.094*	4.2 (2.2–28.0)	0.788	4.0 (0.0–10.1)	*0.097*
Mean oxygen saturation	88.4 ± 3.8	89.0 ± 2.8	0.455	90.5 ± 1.9	0.718	90.9 ± 2.5	*0.004*
Time with oxygen saturation < 90%, min	56.1 (18.9–79.6)	39.5 (24.7–69.8)	1.000	8.9 (5.8–53.6)	0.405	15.0 (5.6–31.5)	*0.044*
Minimum oxygen, %	79.9 ± 7.5	76.3 ± 11.5	0.733	84.3 ± 6.1	0.684	83.7 ± 4.7	0.151
ODI, /h	10.2 (6.2–17.5)	21.5 (11.0–31.0)	0.239	4.7 (0.5–24.8)	0.857	4.8 (2.4–17.3)	0.347
tcCO_2,_ mmHg*	39.9 ± 6.2	40.1 ± 6.6	0.662	42.3 ± 8.4	0.417	38.1 ± 4.9	0.176

Values are mean ± standard deviation or median (interquartile range) for each intervention of about 2 hours in length at night. Italicizised data indicates that the *p* value is below 0.10

*PAP*, Positive airway pressure; *NHF*, nasal high flow; *PH*, precapillary pulmonary hypertension; *TIB*, time in bed; *TST*, total sleep time; *N3*, N3 sleep; *REM*, rapid eye movement sleep; *PLM*, periodic limb movements; *AHI*, apnea-hypopnea index; *AI*, apnea index; *oAI*, obstructive apnea index; *ODI*, oxygen desaturation index. ^**+**^For comparison versus baseline. ^*^Due to artifacts in capnometry recordings data analysis was possible in only 5/6 patients with NHF50

### Impact of NHF50 on sympathovagal balance, sleep, and sleep apnea

NHF50 was poorly tolerated at night, with 6/12 patients refusing therapy and the other six having lower sleep efficiency (Table 3 and Figs. 3 and 4). Compared with no treatment, NHF50 had no significant effects on hemodynamics, SVB (Table 2 and Fig. 2) or OSA severity (Table 3 and Fig. 4). Similar to NHF20, NHF50 had no significant effects on markers of nocturnal SDB severity (Table 3 and Fig. 4).

### Impact of PAP on sympathovagal balance, sleep, and sleep apnea

APAP had no effect on hemodynamic parameters compared with no treatment. The effects of APAP on SVB were heterogenous, with a decrease in the LF/HF ratio of HRV and a trend towards an increase in the LF/HF ratio of dBPV (Table 2 and Fig. 2). APAP had neutral effects on sleep parameters, but significantly improved markers of SDB severity (Table 3 and Figs. 3 and 4).

### Acute impact of NHF20 and NHF50 on sympathovagal balance during daytime

Although NHF20 and NHF50 led to a significant and flow-dependent decrease in subjective well-being (Supplemental Table 1), application of NHF20 and NHF50 during daytime was associated with significant flow-dependent increases in SpO_2_ and to a nonsignificant decrease in tcCO_2_ when compared with no treatment (Supplemental Table 1, Supplemental Figure 1). Furthermore, NHF20 and NHF50 were associated with flow-dependent shift of SVB towards increased sympathetic drive (change in median LF/HF ratio from 0.9 to 1.5 with NHF20 and 1.6 NHF50, respectively; all *p* < 0.05) (Supplemental Table 1, Supplemental Figure 2). NHF20 and NHF50 were also associated with flow-dependent increased measures of cardiac afterload, as documented by the observed increase in diastolic blood pressure and by the slight increase of the total peripheral resistance index (Supplemental Table 1, Supplemental Figure 2).

## Discussion

This is the first study to address the effects of NHF on SVB, sleep, and SDB in adult patients with PH in comparison with PAP therapy. The key findings are as follows: NHF20 was associated with desirable decreases in sympathetic drive during sleep, even in the absence of a significant effect on sleep apnea; PAP therapy slightly improved sleep apnea and significantly improved oxygenation but this does not translate into improved sleep, and effects on SVB were heterogenous; and application of NHF during the day was associated with flow-dependent improvements in gas exchange. However, in contrast to our initial hypothesis, acute application of NHF was associated with flow-dependent undesirable increases in sympathetic drive and a flow-dependent decrease in subjective well-being.

### Impact of NHF20 on sympathovagal balance, sleep, and sleep apnea

Overall, NHF20 decreased indices of sympathetic drive and increased indices of parasympathetic drive, with a trend towards increased sleep efficiency. However, the level of PAP generated during NHF20 was not sufficient to reduce obstructive apneic or hypopneic events in adults with PH. This is consistent with previous studies showing that every 10 L increase in NHF flow rate generates about 1 cm H_2_O of positive airway pressure [36]. Current guidelines recommend treating adults with OSA with 5–12 cm H_2_O of PAP, and NHF20 would likely only produce about 2 cm H_2_O of PAP [11]. However, NHF can have significant effects on obstructive events on children, who require lower PAP levels [17].

Based on the low levels of PAP delivered, the reduced sympathetic drive observed in sleeping PH patients treated with NHF20 can only be explained by the effects on dead space ventilation [20]. It has previously been shown that wasted ventilation (i.e., dead space ventilation) contributes to chronic hyperventilation, dyspnea, and air hunger in patients with PH, causing physical exhaustion and sympathetic overactivity [21, 37]. NHF has been shown to reduce dead space ventilation and make breathing more efficient, as previously demonstrated in ten healthy volunteers using scintigraphy and directly measuring oxygen (O_2_) and CO_2_ in the upper airways [22].

### Impact of NHF50 on sympathovagal balance, sleep, and sleep apnea

We found that NHF50 was poorly tolerated by ambulatory patients with PH. This appears to contradict previous studies showing marked improvement of respiratory parameters and subjective well-being during treatment with NHF50 in patients with chronic obstructive pulmonary disease (COPD), heart failure, or acute hypercapnic respiratory failure [38–42]. In contrast, the patients with stable PH in our study complained about the noise and uncomfortable nasal sensations during use of NHF50. This discomfort was so severe that half of all patients requested to discontinue treatment after several minutes. The fact that sleep efficiency was reduced in the six patients who tolerated use of NHF50 for 2 h reinforces the observation that this flow rate is poorly tolerated at night. However, at a flow rate of 50 L/min, NHF has the potential to generate up to 5 cm H_2_O of PAP, which is at the lower end of the range of pressured recommended for PAP therapy of OSA [11]. Unfortunately, the decreased power resulting from the withdrawal of half the patients during use of NHF50 means that definitive conclusions about PAP delivery and treatment of OSA cannot be made.

### Impact of PAP on sympathovagal balance, sleep, and sleep apnea

As expected, use of PAP improved SDB in patients with PH but did not lead to improved sleep in our study. The effects of PAP therapy on SVB are heterogenous. A previous study in patients with left-sided heart failure showed that PAP may have hypotensive effects [15], leading to unwanted increases in sympathetic drive that may counteract the positive effects associated with reversal of upper airway obstructions [15]. This complex SVB scenario is covered by our data. On the one hand, there is a decrease in the LF/HF ratio based upon HRV analysis (which may reflect the overall picture of SVB best, showing an overall desired decrease in SVB derived from upper airway stability through PAP). On the other hand, there is a trend towards an increase in the LF/HF ratio of dBPV analysis (which may reflect the negative effect of PAP on the right heart due to decreased venous return) [29–31, 33]. It has been speculated that PAP therapy has hypotensive effects in patients with right ventricular dysfunction (with increased sympathetic drive as a response) [15]. Olsson and colleagues showed this in a study on patients with PH [43]. Although the present study did not reveal adverse effects of PAP on blood pressure, the effects of PAP therapy on blood pressure may have been there, which was slightly higher during PAP compared with no treatment. This may apply to automatically titrating PAP therapy (as used in the present study) in particular (compared with continuous PAP therapy, which delivers a more constant and, on average, probably lower level of pressure) [44]. As a result, the present study adds to the growing body of evidence that initiation of PAP therapy in patients with PH and obstructive sleep apnea should be a highly individualized decision.

### Short-term effects of NHF on sympathovagal balance at daytime

Flow-dependent favorable effects of NHF on gas exchange have been previously described [22]. Flow-dependent improvements in oxygenation, reduced anatomical dead space, improved breathing efficiency and generation of PAP have been discussed as potential mechanisms [22]. We hypothesized that clearance of anatomical dead space by NHF would translate into improved wellbeing and a decrease in sympathetic drive in the awake state. In fact, we found the exact opposite, with SVB parameters showing a relative, flow-dependent increase in sympathetic activity. Two main mechanisms may help explain these results. Firstly, the effects of NHF on oxygen saturation may differ between daytime and nighttime. During the day, NHF20 increased mean SpO_2_ from approximately 91 to 93%, while at nighttime, where hypoxia has previously been described to be more severe [8, 10], mean oxygen saturation was approximately 88% in the same patients. Faster and greater increases in oxygen saturation have been shown to be associated with increased levels of oxygen radicals, which in turn are associated with poor functional status in patients with heart failure [43, 45–47]. It appears that differences in the effects of NHF20 on oxygen saturation also account for the unwanted increase in sympathetic drive during daytime and a beneficial reduction in sympathetic drive at night in the present study. Additionally, patient frustrations and lack of tolerance with NHF20 and NHF50 usage during the day may trigger cortical mechanisms of sympathetic activation, overcoming the beneficial effects on dead space. Conversely, this was only true for NHF50 usage at night, whereas NHF20 was better tolerated and beneficial effects on dead space did occur.

### Study limitations

Despite the comprehensive approach taken, our study has limitations that should be considered. Firstly, the sample size was small, and although the study was adequately powered to investigate the effects of NHF and PAP on SVB, it was probably underpowered for assessment of effects on sleep-related breathing. This is particularly true for the effects of NHF50 at night because half of all patients declined treatment. Also in that sense and taking into account the statistical assumption behind the calculation of the Bonferroni post hoc corrections for multiple *t* tests actual *p* values reported in Tables 2 and 3 and Supplemental Table 1 should be interpreted with caution, but statistically significant *p* values on HRV obtained and reported for HRV were still significant even taking into account the Bonferroni post hoc correction

Secondly, the duration of interventions was limited to 2 h, and it is possible that longer periods of NHF may have had different effects on study outcomes. Future studies with longer use of NHF are needed to test the hypothesis that NHF20 favorably alters sympathetic drive and improves the functional status of patients with precapillary PH. Likewise, we do not know whether longer periods of PAP treatment of OSA in PH might decrease sympathetic drive.

Thirdly, sympathetic drive was not measured invasively (e.g., by direct recording of muscle sympathetic nerve activity [MSNA]). Noninvasive recording of HRV and BPV can only provide a general estimation of SVB, which includes the vagal component. However, close correlation between MSNA and HRV/BPV measurements (LF/HF ratios in particular) have previously been shown in healthy volunteers and patients with heart failure [33, 48, 49], and the use of this methodology under different breathing and ventilation-related experimental conditions has previously been established by our group [50].

Fourthly, PAP therapy was not manually titrated to optimal pressures, which may explain why the pressure range used (5–12 cm H_2_O) did not completely normalize the AHI. Ultimately, the results obtained in the present study in patients with PH cannot be applied to different patient populations such as patients with COPD wherein NHF may correct hypoxia and dead space ventilation; the result of which may result in decreased sympathetic drive [51, 52].

Fifthly, another limitation of the study was that the 10 min N2 intervals chosen were not adjusted for respiratory rate. However, as the segments were only 2 h long, it was extremely difficult to find distinct 10 min recordings of constant stable N2 with no artifacts and comparable respiratory rates. Furthermore, in a nighttime setting, it would not have been possible to additionally adjust for respiratory rate as previously established by our group [53, 54].

Finally, scoring of PSG raw data was performed using an unblinded approach regarding the intervention being used (no treatment, NHF20, NHF50, APAP). Ideally, the scorers would have been blinded to this information.

## Conclusion

In conclusion, the effects of NHF on sleep and sympathetic drive in ambulatory patients with precapillary PH and OSA are complex and highly dependent on both the flow rate used and whether the patient is awake or asleep. Desired decreases in sympathetic drive during sleep were achieved with NHF20 (but not NHF50), but this was not the case when patients were awake and fully conscious. The effects of NHF20 on SVB occurred even in the absence of improvements in OSA and are most likely to have occured due to washout of anatomical dead space. In contrast, NHF50 was poorly tolerated by ambulatory patients with PH. Future studies applying long-term use of NHF20 are needed to test whether this intervention favorably alters sympathetic drive and improves the functional status of patients with precapillary PH.

## Electronic supplementary material

ESM 1 (DOCX 94 kb)

ESM 2 (DOCX 73 kb)

ESM 3 (DOCX 20 kb)

## References

[1] Velez-Roa S, Ciarka A, Najem B, Vachiery JL, Naeije R, van de Borne P (2004). Increased sympathetic nerve activity in pulmonary artery hypertension. Circulation.

[2] Hoeper MM, Kramer T, Pan Z (2017). Mortality in pulmonary arterial hypertension: prediction by the 2015 European pulmonary hypertension guidelines risk stratification model. Eur Respir J.

[3] Lammers AE, Munnery E, Hislop AA, Haworth SG (2010). Heart rate variability predicts outcome in children with pulmonary arterial hypertension. Int J Cardiol.

[4] Da Silva Gonçalves Bós D, Van Der Bruggen CEE, Kurakula K (2018). Contribution of impaired parasympathetic activity to right ventricular dysfunction and pulmonary vascular remodeling in pulmonary arterial hypertension. Circulation.

[5] Yi HT, Hsieh YC, Wu TJ, Huang JL, Lin WW, Liang KW, Su CS, Tsai WJ, Wang KY (2014). Heart rate variability parameters and ventricular arrhythmia correlate with pulmonary arterial pressure in adult patients with idiopathic pulmonary arterial hypertension. Heart Lung.

[6] Semen K, Yelisyeyeva O, Jarocka-Karpowicz I, Kaminskyy D, Solovey L, Skrzydlewska E, Yavorskyi O (2016). Sildenafil reduces signs of oxidative stress in pulmonary arterial hypertension: evaluation by fatty acid composition, level of hydroxynonenal and heart rate variability. Redox Biol.

[7] Yoshida K, Saku K, Kamada K, Abe K, Tanaka-Ishikawa M, Tohyama T, Nishikawa T, Kishi T, Sunagawa K, Tsutsui H (2018). Electrical vagal nerve stimulation ameliorates pulmonary vascular remodeling and improves survival in rats with severe pulmonary arterial hypertension. JACC Basic Transl Sci.

[8] Minic M, Ryan CM (2015). Significance of obstructive sleep apnea in the patient with pulmonary hypertension. Curr Opin Pulm Med.

[9] Vitarelli A, Terzano C, Saponara M, Gaudio C, Mangieri E, Capotosto L, Pergolini M, D'Orazio S, Continanza G, Cimino E (2015). Assessment of right ventricular function in obstructive sleep apnea syndrome and effects of continuous positive airway pressure therapy: a pilot study. Can J Cardiol.

[10] Minic M, Granton JT, Ryan CM (2014). Sleep disordered breathing in group 1 pulmonary arterial hypertension. J Clin Sleep Med.

[11] Ramar K, Dort LC, Katz SG, Lettieri CJ, Harrod CG, Thomas SM, Chervin RD (2015). Clinical Practice Guideline for the Treatment of Obstructive Sleep Apnea and Snoring with Oral Appliance Therapy: An Update for 2015. J Clin Sleep Med.

[12] Spicuzza L, Bernardi L, Balsamo R, Ciancio N, Polosa R, di Maria G (2006). Effect of treatment with nasal continuous positive airway pressure on ventilatory response to hypoxia and hypercapnia in patients with sleep apnea syndrome. Chest.

[13] Garet M, Barthélémy JC, Degache F (2006). Modulations of human autonomic function induced by positive pressure-assisted breathing. Clin Physiol Funct Imaging.

[14] Bradley TD, Floras JS (2009). Obstructive sleep apnoea and its cardiovascular consequences. Lancet.

[15] Oldenburg O, Bartsch S, Bitter T, Schmalgemeier H, Fischbach T, Westerheide N, Horstkotte D (2012). Hypotensive effects of positive airway pressure ventilation in heart failure patients with sleep-disordered breathing. Sleep Breath.

[16] Spießhöfer J, Fox H, Lehmann R, Efken C, Heinrich J, Bitter T, Körber B, Horstkotte D, Oldenburg O (2016). Heterogenous haemodynamic effects of adaptive servoventilation therapy in sleeping patients with heart failure and Cheyne–Stokes respiration compared to healthy volunteers. Heart Vessel.

[17] Joseph L, Goldberg S, Shitrit M, Picard E (2015). High-flow nasal cannula therapy for obstructive sleep apnea in children. J Clin Sleep Med.

[18] McGinley B, Halbower A, Schwartz AR, Smith PL, Patil SP, Schneider H (2009). Effect of a high-flow open nasal cannula system on obstructive sleep apnea in children. Pediatrics.

[19] Hawkins S, Huston S, Campbell K, Halbower A (2017). High-flow, heated, humidified air via nasal cannula treats CPAP-intolerant children with obstructive sleep apnea. J Clin Sleep Med.

[20] Robertson HT (2015). Dead space: the physiology of wasted ventilation. Eur Respir J.

[21] Godinas L, Sattler C, Lau EM, Jaïs X, Taniguchi Y, Jevnikar M, Weatherald J, Sitbon O, Savale L, Montani D, Simonneau G, Humbert M, Laveneziana P, Garcia G (2017). Dead-space ventilation is linked to exercise capacity and survival in distal chronic thromboembolic pulmonary hypertension. J Heart Lung Transplant.

[22] Möller W, Feng S, Domanski U, Franke KJ, Celik G, Bartenstein P, Becker S, Meyer G, Schmid O, Eickelberg O, Tatkov S, Nilius G (2017). Nasal high flow reduces dead space. J Appl Physiol.

[23] Fricke K, Schneider H, Biselli P, Hansel N, Zhang Z, Sowho M, Grote L (2018). Nasal high flow, but not supplemental O2, reduces peripheral vascular sympathetic activity during sleep in COPD patients. Int J Chron Obstruct Pulmon Dis.

[24] Galiè N, Humbert M, Vachiery J-L, Gibbs S, Lang I, Torbicki A, Simonneau G, Peacock A, Vonk Noordegraaf A, Beghetti M, Ghofrani A, Gomez Sanchez MA, Hansmann G, Klepetko W, Lancellotti P, Matucci M, McDonagh T, Pierard LA, Trindade PT, Zompatori M, Hoeper M (2015). 2015 ESC/ERS guidelines for the diagnosis and treatment of pulmonary hypertension. Eur Respir J.

[25] Lang RM, Badano LP, Mor V (2015). Recommendations for cardiac chamber quantification by echocardiography in adults: an update from the American Society of Echocardiography and the European Association of Cardiovascular Imaging. J Am Soc Echocardiogr.

[26] Baumgartner H, Falk V, Bax JJ, de Bonis M, Hamm C, Holm PJ, Iung B, Lancellotti P, Lansac E, Rodriguez Muñoz D, Rosenhek R, Sjögren J, Tornos Mas P, Vahanian A, Walther T, Wendler O, Windecker S, Zamorano JL, Roffi M, Alfieri O, Agewall S, Ahlsson A, Barbato E, Bueno H, Collet JP, Coman IM, Czerny M, Delgado V, Fitzsimons D, Folliguet T, Gaemperli O, Habib G, Harringer W, Haude M, Hindricks G, Katus HA, Knuuti J, Kolh P, Leclercq C, McDonagh TA, Piepoli MF, Pierard LA, Ponikowski P, Rosano GMC, Ruschitzka F, Shlyakhto E, Simpson IA, Sousa-Uva M, Stepinska J, Tarantini G, Tchétché D, Aboyans V, Windecker S, Aboyans V, Agewall S, Barbato E, Bueno H, Coca A, Collet JP, Coman IM, Dean V, Delgado V, Fitzsimons D, Gaemperli O, Hindricks G, Iung B, Jüni P, Katus HA, Knuuti J, Lancellotti P, Leclercq C, McDonagh T, Piepoli MF, Ponikowski P, Richter DJ, Roffi M, Shlyakhto E, Simpson IA, Zamorano JL, Kzhdryan HK, Mascherbauer J, Samadov F, Shumavets V, Camp GV, Lončar D, Lovric D, Georgiou GM, Linhartova K, Ihlemann N, Abdelhamid M, Pern T, Turpeinen A, Srbinovska-Kostovska E, Cohen A, Bakhutashvili Z, Ince H, Vavuranakis M, Temesvári A, Gudnason T, Mylotte D, Kuperstein R, Indolfi C, Pya Y, Bajraktari G, Kerimkulova A, Rudzitis A, Mizariene V, Lebrun F, Demarco DC, Oukerraj L, Bouma BJ, Steigen TK, Komar M, de Moura Branco LM, Popescu BA, Uspenskiy V, Foscoli M, Jovovic L, Simkova I, Bunc M, de Prada JAV, Stagmo M, Kaufmann BA, Mahdhaoui A, Bozkurt E, Nesukay E, Brecker SJD, ESC Scientific Document Group (2017). 2017 ESC/EACTS Guidelines for the management of valvular heart disease. Eur Heart J.

[27] Maceira AM, Prasad SK, Khan M, Pennell DJ (2006). Reference right ventricular systolic and diastolic function normalized to age, gender and body surface area from steady-state free precession cardiovascular magnetic resonance. Eur Heart J.

[28] Berry RB, Budhiraja R, Gottlieb DJ, Gozal D, Iber C, Kapur VK, Marcus CL, Mehra R, Parthasarathy S, Quan SF, Redline S, Strohl KP, Davidson Ward SL, Tangredi MM, American Academy of Sleep Medicine (2012). Rules for scoring respiratory events in sleep: update of the 2007 AASM Manual for the Scoring of Sleep and Associated Events. Deliberations of the Sleep Apnea Definitions Task Force of the American Academy of Sleep Medicine. J Clin Sleep Med.

[29] Fortin J, Habenbacher W, Heller A, Hacker A, Grüllenberger R, Innerhofer J, Passath H, Wagner C, Haitchi G, Flotzinger D, Pacher R, Wach P (2006). Non-invasive beat-to-beat cardiac output monitoring by an improved method of transthoracic bioimpedance measurement. Comput Biol Med.

[30] Fortin J, Marte W, Grüllenberger R, Hacker A, Habenbacher W, Heller A, Wagner CH, Wach P, Skrabal F (2006). Continuous non-invasive blood pressure monitoring using concentrically interlocking control loops. Comput Biol Med.

[31] Gratze G, Fortin J, Holler A, Grasenick K, Pfurtscheller G, Wach P, Schönegger J, Kotanko P, Skrabal F (1998). A software package for non-invasive, real-time beat-to-beat monitoring of stroke volume, blood pressure, total peripheral resistance and for assessment of autonomic function. Comput Biol Med.

[32] Karemaker JM (2017). An introduction into autonomic nervous function. Physiol Meas.

[33] Pagani M, Montano N, Porta A, Malliani A, Abboud FM, Birkett C, Somers VK (1997). Relationship between spectral components of cardiovascular variabilities and direct measures of muscle sympathetic nerve activity in humans. Circulation.

[34] Mirizzi G, Giannoni A, Bramanti F, Ripoli A, Varanini M, Bernardi L, Emdin M, Passino C (2013). Letter to the Editor a simple method for measuring baroreflex sensitivity holds prognostic value in heart failure. Int J Cardiol.

[35] Berry RB, Gamaldo CE, Harding SM, Lloyd RM, Marcus CL, Vaughn BV for the American Academy of Sleep Medicine BR (2015). The AASM manual for the scoring of sleep and associated events: rules, terminology and technical specifications, Version 2.2. J Clin Sleep Med.

[36] Rarle RL, Bloch A, Mcguinness SP, Chb MB (2015). Effect of very-high-flow nasal therapy on airway pressure and end-expiratory lung impedance in healthy volunteers. Respir Care.

[37] Hoeper MM, Pletz MW, Golpon H, Welte T (2007). Prognostic value of blood gas analyses in patients with idiopathic pulmonary arterial hypertension. Eur Respir J.

[38] Díaz-Lobato S, Folgado MA, Chapa A, Mayoralas Alises S (2013). Efficacy of high-flow oxygen by nasal cannula with active humidification in a patient with acute respiratory failure of neuromuscular origin. Respir Care.

[39] Bräunlich J, Köhler M, Wirtz H (2016). Nasal highflow improves ventilation in patients with COPD. Int J COPD.

[40] Millar J, Lutton S, O’Connor P (2014). The use of high-flow nasal oxygen therapy in the management of hypercarbic respiratory failure. Ther Adv Respir Dis.

[41] Carratalá JM, Díaz Lobato S, Brouzet B, Más-Serrano P, Espinosa B, Llorens P (2018). Efficacy and safety of high-flow nasal cannula oxygen therapy in patients with acute heart failure. Emergencias.

[42] Kang MG, Kim K, Ju S, Park HW, Lee SJ, Koh JS, Hwang SJ, Hwang JY, Bae JS, Ahn JH, Jang JY, Park Y, Jeong YH, Kwak CH, Park JR (2019). Clinical efficacy of high-flow oxygen therapy through nasal cannula in patients with acute heart failure. J Thorac Dis.

[43] Olsson KM, Frank A, Fuge J, Welte T, Hoeper MM, Bitter T (2015). Acute hemodynamic effects of adaptive servoventilation in patients with pre-capillary and post-capillary pulmonary hypertension. Respir Res.

[44] Karasulu L, Epöztürk PÖ, Sökücü SN, Dalar L, Altın S (2010). Improving heart rate variability in sleep apnea patients: differences in treatment with auto-titrating positive airway pressure (APAP) versus conventional CPAP. Lung.

[45] Fujita N, Yamasaki N, Eto K, Asaeda M, Kuwahara W, Imagita H (2018). Oxygen therapy may worsen the survival rate in rats with monocrotaline-induced pulmonary arterial hypertension. PLoS One.

[46] Kim TY, Kim DH, Kim SC, Kang C, Lee SH, Jeong JH, Lee SB, Park YJ, Lim D (2018). Impact of early hyperoxia on 28-day in-hospital mortality in patients with myocardial injury. PLoS One.

[47] Wedgwood S, Steinhorn RH, Lakshminrusimha S (2019). Optimal oxygenation and role of free radicals in PPHN. Free Radic Biol Med.

[48] Van de Borne P, Montano N, Zimmerman B (1997). Relationship between repeated measures of hemodynamics, muscle sympathetic nerve activity, and their spectral oscillations. Circulation.

[49] Kienzle MG, Ferguson DW, Birkett CL, Myers GA, Berg WJ, Mariano DJ (1992). Clinical, hemodynamic and sympathetic neural correlates of heart rate variability in congestive heart failure. Am J Cardiol.

[50] Spiesshoefer J, Becker S, Tuleta I, Mohr M, Diller GP, Emdin M, Florian AR, Yilmaz A, Boentert M, Giannoni A (2019). Impact of simulated hyperventilation and periodic breathing on sympatho-vagal balance and hemodynamics in patients with and without heart failure. Respiration.

[51] Biselli P, Fricke K, Grote L et al (2018) Reductions in dead space ventilation with nasal high flow depend on physiological dead space volume: metabolic hood measurements during sleep in patients with COPD and controls. Eur Respir J 51 10.1183/13993003.02251-2017 29724917

[52] Biselli PJ, Kirkness JP, Grote L (2016). Nasal high-flow therapy reduces work of breathing compared with oxygen during sleep in COPD and smoking controls: a prospective observational study. J Appl Physiol (1985).

[53] Gorbachevski M, Spiesshoefer J, Arzt M, Oldenburg O, Becker S, Tuleta I, Emdin M, Passino C, Sciarrone P, Boentert M, Giannoni A (2020) Adaptive servo-ventilation therapy does not favourably alter sympatho-vagal balance in sleeping patients with systolic heart failure and central apnoeas: preliminary data. Int J Cardiol. 10.1016/j.ijcard.2020.03.078 Online ahead of print 10.1016/j.ijcard.2020.03.078 32317236

[54] Spiesshoefer J, Hegerfeld N, Gerdes MF, Klemm S, Gorbachevski M, Radke R, Tuleta I, Passino C, Jiang X, Sciarrone P, Randerath W, Dreher M, Boentert M, Giannoni A (2020). Effects of central apneas on sympathovagal balance and hemodynamics at night: impact of underlying systolic heart failure. Sleep Breath.

