# Supplementary figures and images for: Effects of nasal high flow on sympathovagal balance, sleep, and sleep-related breathing in patients with precapillary pulmonary hypertension
Source: Sleep Breath. 2020 Aug 22;25(2):705–17. doi: 10.1007/s11325-020-02159-1 (PMC8195975; doi:10.1007/s11325-020-02159-1)

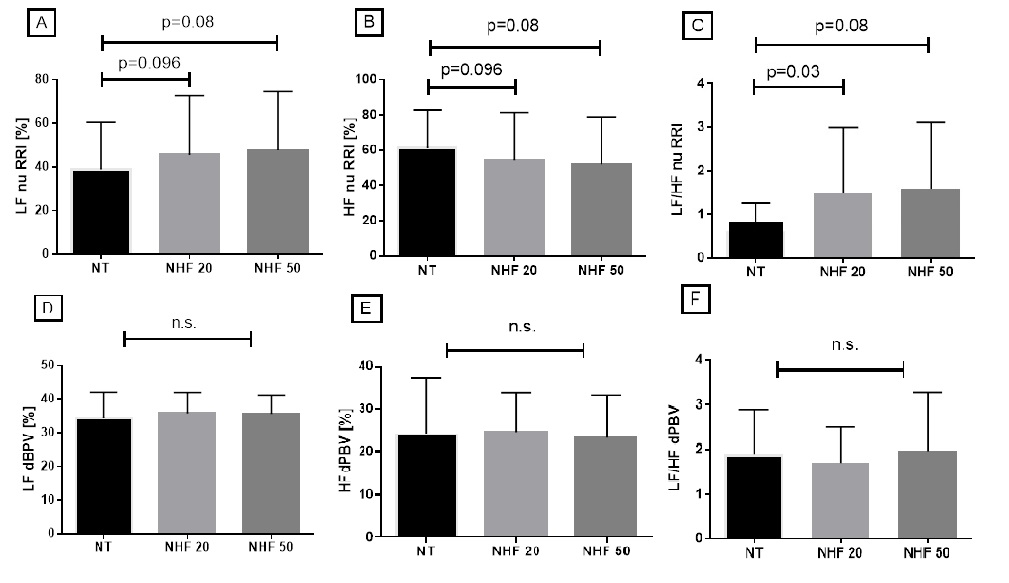

Supplement: Supplementary file 1 — (DOCX 94 kb) [file 11325_2020_2159_MOESM1_ESM.docx]

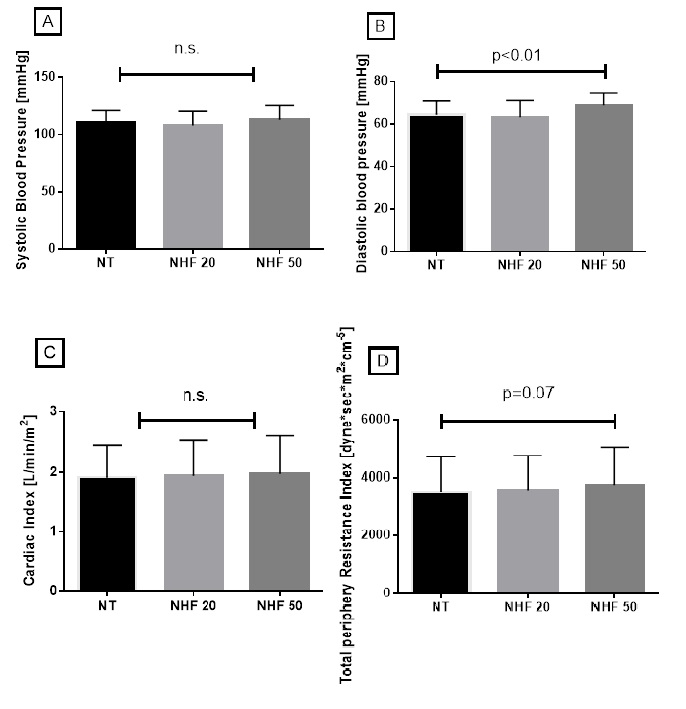

Supplement: Supplementary file 2 — (DOCX 73 kb) [file 11325_2020_2159_MOESM2_ESM.docx]
