# Supplementary material for: Effects of nasal high flow on sympathovagal balance, sleep, and sleep-related breathing in patients with precapillary pulmonary hypertension
Source: Sleep Breath. 2020 Aug 22;25(2):705–17. doi: 10.1007/s11325-020-02159-1 (PMC8195975; doi:10.1007/s11325-020-02159-1)
Supplement: Supplementary file 3 — (DOCX 20 kb) [file 11325_2020_2159_MOESM3_ESM.docx]

***Online Supplement***

***Effects of Nasal High Flow on Sympathovagal Balance, Sleep and Sleep-***

***related Breathing in Patients with Precapillary Pulmonary***

***Hypertension***

Jens Spiesshoefer^1,2#*^, Britta Bannwitz^1#^, Michael Mohr^3^, Simon Herkenrath^4^, Winfried Randerath^4^,

Paolo Sciarrone^5^, Christian Thiedemann^1^, Hartmut Schneider^6^, Andrew T. Braun^7^, Michele Emdin^2,5^,

Claudio Passino^2,5^, Matthias Boentert^1×^, Alberto Giannoni^2,5×^

^1^ Department of Neurology with Institute for Translational Neurology, University of Muenster,

Muenster, Germany

^2^ Institute of Life Sciences, Scuola Superiore Sant´Anna, Pisa, Italy

^3^ Department of Medicine A, Hematology, Oncology and Pulmonary Medicine, University Hospital

Muenster, Muenster, Germany

^4^ Bethanien Hospital gGmbH Solingen, Solingen, Germany and Institute for Pneumology at the

University of Cologne, Solingen, Germany

^5^ Cardiology and Cardiovascular Medicine Division, Fondazione Toscana Gabriele Monasterio,

National Research Council, CNR-Regione Toscana, Pisa, Italy

^6^ Sleep Disorders Center, Bayview Hospital, School of Medicine, Johns Hopkins University, Baltimore,

USA

^7^ Division of Allergy, Pulmonary and Critical Care, Department of Medicine, University of Wisconsin,

Madison, USA

**Supplemental Table 1.** Effect of nasal high flow therapy on sympathovagal balance and hemodynamic parameters during wakefulness

|  | **Baseline** | **NHF20** | **P-value^+^** | **NHF50** | **P-value^+^** |
| --- | --- | --- | --- | --- | --- |
| **Wellbeing** (0-10) | 7.7±2.3 | 6.7±2.9 | **0.047** | 5.4±2.9 | **0.007** |
| **Respiratory parameters**  Mean oxygen saturation, %  tcCO_2_, mmHg* | 90.9±4.5  37.2±2.6 | 92.8±3.4  35.5±4.0 | **0.026**  0.250 | 93.3±2.4  34.9±4.7 | **0.056**  0.074 |
| **Sympathovagal balance parameters** | |  |  |  |  |
| HFnuRRI, % | 61.1±21.7 | 54.4±27.1 | **0.096** | 52.1±26.8 | **0.080** |
| LFnuRRI, % | 38.9±21.7 | 45.6±27.1 | **0.096** | 47.9±26.8 | **0.080** |
| LF/HF nu RRI | 0.9 ± 1.0 | 1.5 ± 1.6 | **0.031** | 1.6 ± 1.6 | **0.077** |
| HF dBPV, mmHg² | 24.2±12.8 | 24.7±9.2 | 0.855 | 23.4±9.8 | 0.826 |
| LF dBPV, mmHg² | 34.4±7.6 | 35.7±6.2 | 0.588 | 35.6±5.6 | 0.675 |
| LF/HF nu dBPV | 1.9± 1.0 | 1.7 ± 0.8 | 0.415 | 1.9 ± 1.3 | 0.881 |
| BRS Slope** |  |  |  |  |  |
| Up-event count, n | 8.5 (4.8-19.0)^^^ | 11.0 (10.5-24.0)^^^ | 0.302 | 24.5 (17.0-31.0)^^^ | **0.013** |
| Up-events, ms/mmHg | 12.3 (7.5-19.1)^^^ | 8.1 (6.2-11.1)^^^ | **0.008** | 6.7 (5.8-12.9)^^^ | **0.008** |
| Down-event count, n | 11.0 (10.0-14.3)^^^^ | 7.5 (5.3-11.8)^^^^ | 0.717 | 10.0 (6.3-19.3)^^^^ | 0.770 |
| Down-events, ms/mmHg | 8.8 (6.6-10.9)^^^^ | 9.1 (6.4-11.9)^^^^ | 0.375 | 7.8 (5.3-15.0)^^^^ | 0.105 |
| **Hemodynamic parameters** |  |  |  |  |  |
| Heart rate, min^-1^ | 69.1±9.5 | 68.7±9.7 | 0.520 | 69.2±10.4 | 0.890 |
| Systolic BP, mmHg | 111.1±10.3 | 108.2±12.3 | 0.345 | 113.1±12.5 | 0.587 |
| Diastolic BP, mmHg | 64.5±6.5 | 63.2±8.0 | 0.204 | 68.9±5.8 | **0.009** |
| Stroke volume index, mL/m² | 27.3±5.7 | 28.1±6.4 | 0.104 | 28.3±6.6 | 0.104 |
| Cardiac index, L/min/ m² | 1.9±0.5 | 1.9±0.6 | 0.266 | 2.0±0.6 | 0.297 |
| TPRI, dyne·sec·m²·cm^-5^ | 3490.7±1259.0 | 3548.9±1231.0 | 0.622 | 3755.3±1279.8 | **0.074** |

Values mean ± standard deviation or median (interquartile range) for segments of 10 minutes duration taken at daytime and in sinus rhythm. PAP, positive airway pressure; NHF20, nasal high flow therapy at 20 L/min; NHF50, nasal high flow therapy at 50 L/min; PH, precapillary pulmonary hypertension; BRS Slope, slope of baroreceptor reflex sensitivity (up events and down events); HFnudBPV high frequency component of diastolic blood pressure variability; HFnuRRI, high frequency component of heart rate variability; LFnudBPV, low frequency component of diastolic blood pressure variability; LF/HF dBPV, relative ratio of low frequency and high frequency component of diastolic blood pressure variability; LFnuRRI, low frequency component of heart rate variability; LF/HF RRI, relative ratio of low frequency and high frequency component of heart rate variability; nu, normalized units (normalised for total power spectra); for both measures a higher ratio reflects increased sympathetic drive as LF component (of diastolic blood pressure variability in particular) is believed to reflect sympathetic drive and HF component (of both HRV and dBPV) is believed to reflect parasympathetic drive; TPRI, total peripheral resistance index. **^+^**For comparison versus baseline. ^*^Due to artifacts in the capnometry readings analysis was possible in 9/12 patients.^**^Due to the low number of events during the segment chosen analysis of BRS was made in 8/12 patients for up-events, and 10/12 patients for down-events.

**Supplemental Figure Legends**

**Supplemental Fig. 1.** Impact of nasal high flow at 20 L/min (NHF20) and nasal high flow at 50 L/min (NHF50) compared with no treatment (NT) on sympathovagal balance: low frequency component of heart rate variability in units normalized for total power spectra [LFnuRRI] (**A**), : high frequency component of heart rate variability in units normalized for total power spectra [HFnuRRI] (**B**), LF/HFnu(RRI) (**C**), low frequency component of diastolic blood pressure variability in units normalized for total power spectra [LFdBPV] (**D**), high frequency component of diastolic blood pressure variability in units normalized for total power spectra [HFdBPV] (**E**), LF/HFdBPV (**F**). Values are mean ± standard deviation for each 10-minute assessment period.

**Supplemental Fig. 2.** Impact of nasal high flow at 20 L/min (NHF20) and nasal high flow at 50 L/min (NHF50) compared with no treatment (NT) on hemodynamics. Systolic blood pressure (**A**), diastolic blood pressure (**B**), cardiac index (**C**) and total peripheral resistance index (**D**). Values are mean ± standard deviation for each 10-minute assessment period.
